# Water uptake by indoor surface films

**DOI:** 10.1038/s41598-019-47590-x

**Published:** 2019-07-31

**Authors:** Heather Schwartz-Narbonne, D. James Donaldson

**Affiliations:** 1 Department of Chemistry, University of Toronto, 80 St., George St., Toronto, Ontario M5S 3H6 Canada; 2 Department of Physical and Environmental Sciences, University of Toronto, Scarborough 1265 Military Trail, Toronto, Ontario M1C 1A4 Canada

**Keywords:** Chemistry, Environmental chemistry

## Abstract

Indoor surfaces provide a plentiful and varied substrate on which multiphase reactions can occur which can be important to the chemical makeup of the indoor environment. Here, we attempt to characterise real indoor surface films via water uptake behaviour and ionic composition. We show that water uptake by indoor films is different than that observed outdoors, and can vary according to room use, building characteristics, and season. Similarly, preliminary investigation into the ionic composition of the films showed that they varied according to the room in which they were collected. This study highlights the importance of different types of soiling to multiphase chemistry, especially those reactions controlled by relative humidity or adsorbed water.

## Introduction

Concentrations of trace gases in the indoor atmosphere are controlled primarily by ventilation, human occupancy/activity, and indoor chemical reactions. The fact that indoor environments contain high surface area to volume ratios (between 2.9–4.6 m^2^ per m^3^ for residential rooms)^1^ means that heterogeneous chemical reactions have the potential to play a large role in indoor chemistry. A reaction taking place in the condensed phase or on a surface can exhibit a new reactive pathway that reduces its energy requirement. Thus indoor atmospheric chemistry could display quite different reactivity from that observed outdoors.

The important classes of heterogeneous surface chemistry are sorptive interactions, acid–base chemistry, and oxidative reactions^2^. Indoor surfaces are composed of a variety of materials, including gypsum board, paint, glass, wood, tile, laminate, carpet, and fabric. Some of these materials have irregular surfaces and provide large surface areas as well as different sorptive properties^3–15^. When clean, these surfaces vary significantly in their sorptive properties for gas-phase molecules, depending on surface characteristics such as morphology, hydrophobicity/philicity, or pH^3–15^. However, it has been observed that after soiling of different surfaces in a kitchen environment, the partition coefficients for di-2-ethylhexyl phthalate to those surfaces were almost identical, lying close to that of octanol/air^11^. This implies that the materials deposited on indoor surfaces, producing a surface film, can display sorptive properties towards reagents that may be quite different from those of the underlying substrate^11^.

Indoor surface films are complex and dynamic, as their constituents are determined by both the deposition and surface reaction of atmospheric compounds^4,8,9,11,15–32^. They are comprised of an organic portion (that may contain PBDEs, PCBs, PAHs, OCPs, NFRs, phthalates, aldehydes, squalene and other lipids, and fatty acids)^8,9,11,16,18–21,23,25–27,30–34^, and an inorganic portion (containing metals, elemental carbon, water, and ions)^22,23,28,30^. Indoor surface films contain a higher percentage of organic matter content by mass than their outdoor counterparts; this has been attributed to additional indoor sources of organics such as occupants, activities such as cooking, and plasticizers from items within the building^9,10,15,16,24,29,30,32^. Other factors which have been found to affect the composition of indoor surface films are the airflow in a building, the type of glass used for the windows, the distance from a major road, the age of the building, the distance from industrial activity, and occupant activities such as smoking or cleaning^23,26,35^. A study of indoor films collected in a copier room, office, kitchen, and garage showed significant differences in morphology and composition between coatings, attributed to room use^30^. For example, a significant increase in surface roughness due to organic film coverage was observed for coatings collected in the kitchen, and enhanced levels of copper, barium, and zinc were observed in samples collected in the copier room^30^.

Indoor surfaces, especially those in rooms with high relative humidity such as bathrooms or kitchens, could be covered with a thin layer of water^4^. Adsorbed water could have an effect on the sorptive properties of the underlying surface, as the water layer may be more hydrophilic than the material underneath. The presence of water may also facilitate chemical reactions on the surface such as acid-base chemistry, as ions produced from these reactions are stable in the polar water environment. Gases such as CO_2_ and NH_3_ dissolve in water, have significant acid-base potential, and are prevalent indoors^4,12,13,36,37^. The pH of water monolayers controlled by these gases may then alter the ability of organic pollutants to sorb into or desorb from surfaces, and thus their thermodynamic likelihood of reactions^2,4,12,13^. Finally, adsorbed water can affect the oxidative potential of surfaces, acting as a source of hydroxyl radicals, participating in hydrolysis, or blocking photoactive sites on photocatalytic materials^38–45^.

In the present study a quartz crystal microbalance (QCM) was used to measure the water uptake behaviour as a function of ambient relative humidity (RH) of real indoor films collected in different dwellings, seasons, and rooms. An ion chromatograph was used to probe the chemical composition of these films in different rooms. The principle of such measurements using a QCM is that as the mass of deposited water increases, the resonant crystal frequency decreases in a linear manner, allowing sensitive and precise determinations of the mass of water deposited as a function of RH. In previous studies, we have used this method to investigate the water uptake of thin films, including oxidised organic molecules^46,47^, vacuum grease^48^, and outdoor grime^49^ as a function of ambient relative humidity. Such studies can help to elucidate the role adsorbed water plays in chemistry on those surfaces. It is found that in general, as relative humidity in a chamber increases, the mass of water on the surface of the crystal also increases, with the overall water uptake by the film and the shape of the water uptake curve being strongly affected by the hydrophilicity of film constituents^46–49^. For example, Demou *et al*. measured the water uptake of thin organic films of dodecane, 1-octanol, octanoic acid, 1,5-pentanediol, 1,8- octanediol, and malonic acid as a function of relative humidity^47^. More oxidised compounds showed greater sorption of water over the same relative humidity range as compared to their less-oxidised counterparts, which displayed adsorptive as opposed to absorptive water uptake^47^. Compounds which were water soluble solids at room temperature behaved similarly to inorganic salts, showing significant water-uptake, hysteresis, and even deliquescence at high relative humidities^47^. In a later study, urban grime collected outdoors showed very little hysteresis in its water uptake, but the total mass of water on the surface was higher for the grime than for vacuum grease^49^. This was explained by oxidised organic components of the grime as well as deposited ions, however there was no evidence of efflorescence or deliquescence of compounds within the film, in spite of a significant presence of inorganic salts^49^. A more recent study connected water uptake by urban grime to seasonality^50^. Water uptake was highest in the summer, followed by the spring, and it was lowest in the winter. This was postulated to be due to changes in the grime composition, as water uptake scaled with the concentration of oxalate and also with the nitrate to sulfate ratio within the grime. Interestingly, the water uptake did not appear to correlate with the other inorganic ions measured in the film.

## Results and Discussion

### Water uptake by indoor films

**Figure 1:**
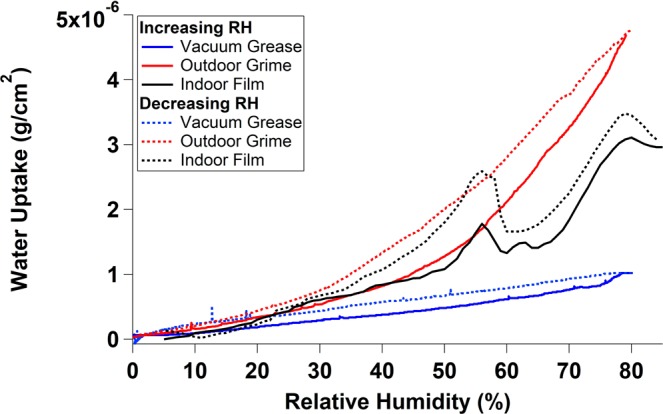
Representative water uptake curves of vacuum grease in blue (collected in 2016 by Clouthier)^48^, outdoor grime in red (collected in December 2014 by Baergen and Donaldson)^50^, and an indoor film in black (collected in July – September as a part of this study). The solid lines indicate increasing relative humidity and dashed lines indicate decreasing relative humidity.

Figure 1 shows representative water uptake as a function of relative humidity for films of vacuum grease (a mixed-hydrocarbon proxy for the organic portion of urban grime^48^) in blue, outdoor urban grime^50^ in red, and an indoor film in black. The methodology for collection and measurement of the grease and outdoor grime samples can be found in the respective publications. For each of the cases illustrated, the solid trace represents the mass gain by the sample as the ambient relative humidity is increased, and the dashed trace shows the corresponding water mass as a function of decreasing RH. The curves pictured in Fig. 1 were not blank-subtracted, and show some hysteresis - that is, a difference in the uptake behavior between increasing and decreasing RH - which was not observed in the blank-subtracted samples. There are clear differences between the three samples pictured here.

Each curve pictured is the result of averaging the water uptake over multiple increasing and decreasing relative humidity cycles. While the water uptake of each cycle was somewhat different in magnitude, the shapes of the curves were constant from run to run. Figure S1 in the Supplementary Information illustrates this reproducibility using a representative indoor film sample and blank. As this level of agreement was observed for all samples, we show in the figures below the results of averaging water uptake curves for exposed crystals and then subtracting the average of all the blank experiments for the crystal in question.

The differences in water uptake behaviour between representative outdoor urban grime, vacuum grease, and indoor surface film samples (as illustrated in Fig. 1) are likely governed by their different chemical compositions, and how these influence their relative hydrophobicities. The vacuum grease used was composed of unsaturated hydrocarbons, and as such was extremely hydrophobic and took up relatively little water. By contrast, the outdoor grime sample contained a mix of organic species (of varying oxidation states), metals, and ions, and so was clearly more hydrophilic than the grease sample, and therefore took up more water across the same relative humidity range^20,23,26,28,29,31,50–59^. The indoor surface film sample is expected to contain a larger organic fraction than its outdoor counterpart, due to variations in room use and ventilation leading to greater accretion and less loss of organics from the surface^8–11,15,16,18–21,23–32,34^ However, as displayed in Table 1, the indoor surface films still contain metals, ions, and oxidised organic species, whose presence is expected to increase their hydrophilicity above that of pure vacuum grease, and may explain the placement of the indoor surface film water uptake curve between that of grease and grime^8,16,18–23,25–30,32–34^.

**Table 1:** Concentrations of ions in indoor films collected in Location 1 between November 19th, 2018 and February 6th, 2019.

Sample	NO_3_^−^	SO_4_^2−^	C_2_O_4_^2−^	Cl^−^	Na^+^	Ca^2+^	NH_4_^+^
**Concentration (ug/cm**^2^**)**							
Living Room	7.57	17.00	23.05	2412.96	3003.72	1272.08	0
Kitchen	25.17	38.72	37.64	2633.24	3184.44	1298.52	0
Kitchen (Extraction 2)	0	0	3.17	0	0	207.90	0

The most striking feature of the water uptake curves of the indoor surface films is their shape. Whereas outdoor urban grime and vacuum grease show smooth water uptake across the RH region of interest, several of the indoor films showed marked departures from this. Peaks in the water uptake were observed when the relative humidity in the chamber reached certain values, such as 56% and 78% in the case of the sample shown in Fig. 1. This implies that, for this sample, as the relative humidity in the chamber increased from 53% to 59% the mass of water adsorbed to the crystal decreased. This behaviour was repeated upon decreasing the relative humidity between 59 and 53%, and the mass of adsorbed water seemed to increase between those values, then decrease monotonically again.

This behaviour is likely a consequence of changes in how the mixture of water and indoor surface film on the QCM crystal surface is coulped to the oscillations of the crystal as the quantity of water on the surface changes^60^. One explanation involves changes in the morphology of the water present on the surface as the humidity is varied. Rodhal *et al*. observed decreases in the QCM frequency as RH was increased, giving water absorption onto the crystal, until about 85% RH, at which point a monolayer of water was formed^60^. From that point on any additional water adsorption added to the total mass of the film but not to the coupling of the oscillations of the film and the crystal; indeed they observed an increase in crystal frequency, which naively suggests a loss of water mass^60^. At a relative humidity of 95% another sudden increase in frequency was observed, which could again be interpreted as a decrease in mass, but which the authors attributed to a 2D-3D shift in the water layer as it broke up into droplets and decreased the coupling between the water and the underlying crystal^60^. Since indoor films are heterogeneous, and such surfaces could lead to heterogeneous water uptake^30^, “pools” of water formed on the QCM surface could lead to fluctuations in the coupling between the crystal and the adsorbed water. Furthermore, real indoor surfaces are often far rougher and more heterogeneous than the QCM crystals used in this study. This morphology could synergise with heterogenous surface films to promote the formation of these pools.

Another possibility is that the peaks arise due to phase changes within the grime as a function of RH, in particularly the deliquescence and efflorescence of ions or oxidized organics. For example, the hydration of NaCl on a crystal surface has been observed to cause an increase in QCM vibrational frequency, (which may be naively interpreted as a decrease in total mass) despite the physical increase in mass due to water adsorption^60^. QCM oscillations only travel in a material for a distance of ~2δ, where δ represents the decay length of the fluid shear wave (the distance at which the wave has decreased by a factor of e). Samples of viscous fluids with thickness greater than 2δ thus give rise to a QCM response equivalent to submersion of the crystal in infinite liquid^60^. In this way, when a layer of a solid material becomes a thick layer of viscous liquid, it is possible to infer the oscillation frequency increase as a decrease in adsorbed mass, because only part of the liquid layer is probed^60^. Figure S2 in the Supplementary Information illustrates the physical situations described above.

We have conducted a few preliminary tests in order to determine if these behaviours could be explained by substances probably present in the indoor surface environment, and have measured the water uptake of skin oil, skin oil with added sodium chloride, and skin oil with added calcium chloride. The results of these tests are displayed in Fig. S3 in the Supplementary Information. While each of these added components produced different water uptake curves, including a clear deliquescence of the NaCl-containing film, none of them exhibited local maxima ("peaks") like those observed in the indoor film samples.

**Figure 2:**
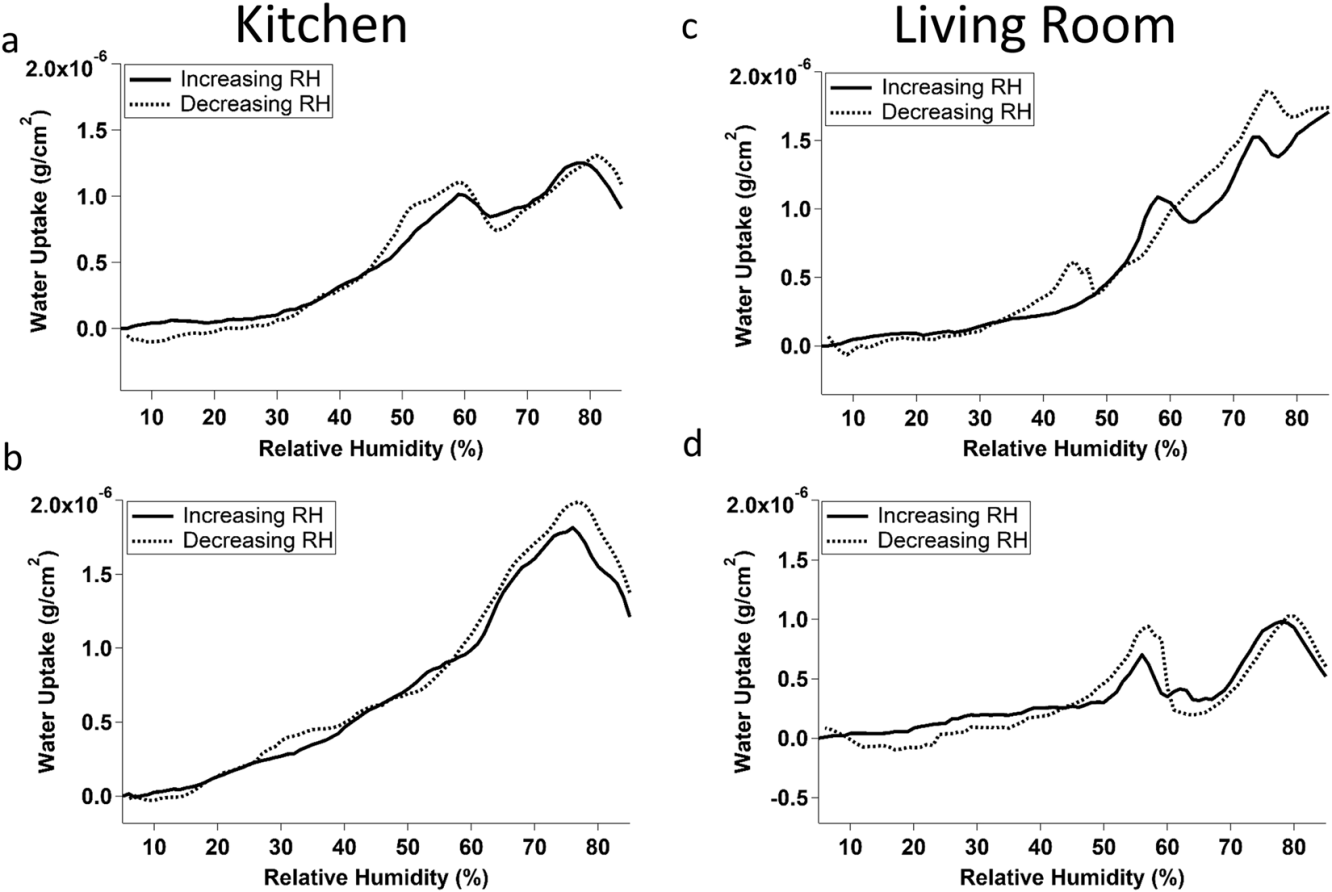
Averaged water uptake curves for 4 film samples collected in the kitchen (**a**,**b**) and living room (**c**,**d**) of Location 1 between July 25, 2018 and September 21, 2018. The solid lines indicate increasing relative humidity and dashed lines indicate decreasing relative humidity.

Figure 2 shows the water uptake of 4 different crystals deployed between July 25^th^ 2018 – September 21^st^, 2018 in Location 1. Two crystals were deployed in a kitchen (panels A and B) and two were deployed in a living room (panels C and D). Each of the four crystals shows water uptake with relatively little hysteresis, as was observed in the outdoor grime and vacuum grease samples (see Fig. 1). However, unlike those other types of film, none of the indoor films showed a smooth water uptake curve. Peaks were observed around 58% RH on samples A, C, and D, and around 77% RH on samples A, B, and D. Sample C showed additional peaks around 43% and 72% RH, and was the only sample where the peaks were different during the humidification phase than the drying phase. The peak at 58% RH was only present when the RH was increasing, while the peak at 43% RH was only present when the RH was decreasing.

**Figure 3:**
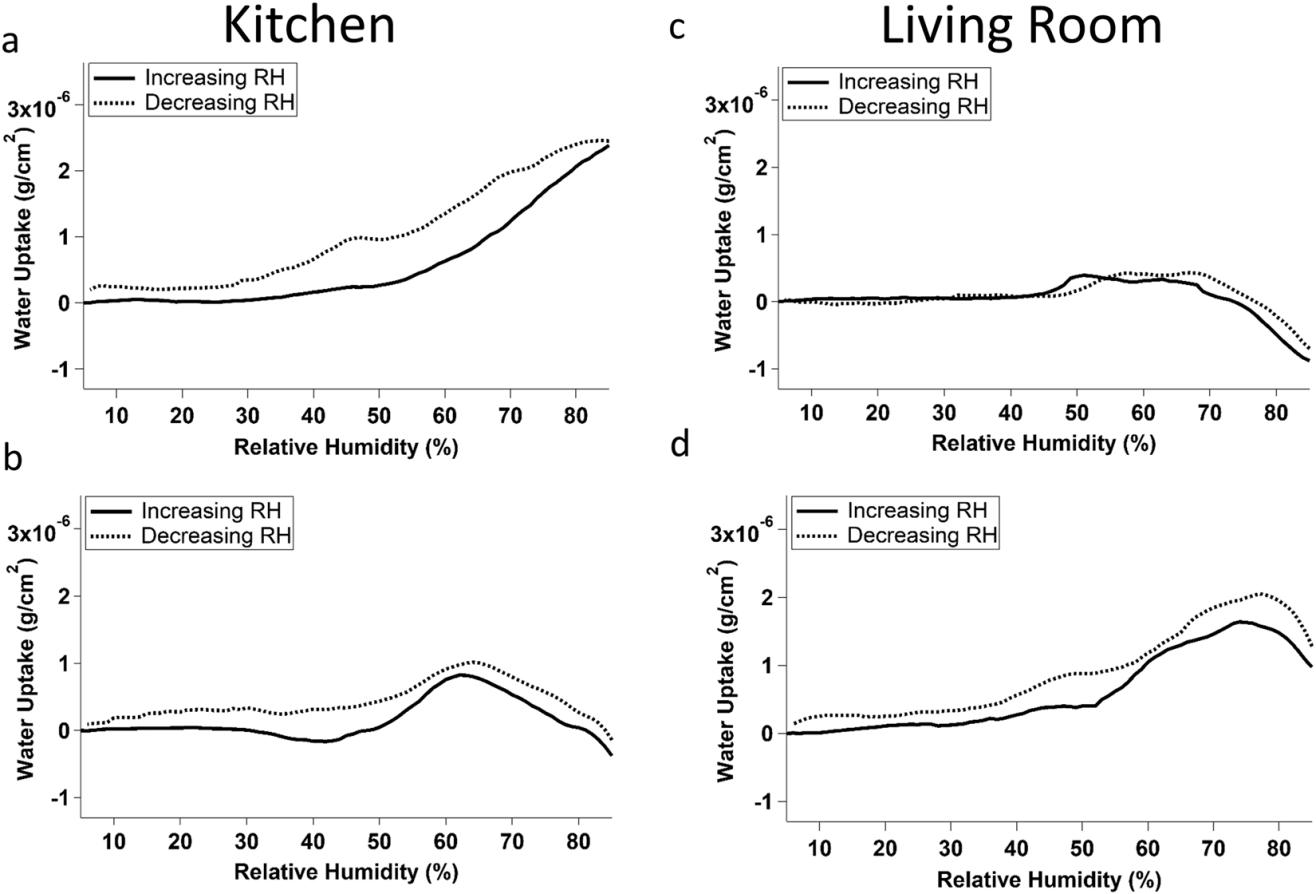
Averaged water uptake curves for 4 film samples collected in the kitchen (**a**,**b**) and living room (**c**,**d**) of Location 1 between October 11, 2018 and December 11, 2018. Solid lines indicate increasing relative humidity and dashed lines indicate decreasing relative humidity.

Water uptake curves of samples collected between October 11^th^ – December 11^th^, 2018, also in Location 1, are displayed in Fig. 3. These curves show greater variation than those collected during sampling period 1. The sample shown in Fig. 3a had an almost completely smooth uptake curve, with greater hysteresis than was observed in any other sample. The samples illustrated in Fig. 3b–d showed very little water uptake and little hysteresis across the humidity range studied. Peaks were observed in samples 4B and 4D at 62% and 75% RH respectively. The samples in Fig. 3b,c were particularly striking, as they showed an apparent overall negative water uptake. Because all the curves are blank subtracted, a negative value represents a smaller water uptake than was observed when the experiment was conducted using a clean crystal (but note the above discussion concerning apparent mass decreases during water uptake).

Comparing Figs 2 and 3 allows us to examine water uptake across living room and kitchen environments in Location 1 during two different sampling periods: July 25, 2018 - September 21, 2018 (period 1) and October 11, 2018 - December 11, 2018 (period 2). In both figures there is no significant variation in water uptake depending on the sampling room. However, there is a notable difference between the samples collected during the first period and those collected during the second period. The samples collected during the first period all showed an increase in mass over the relative humidity range tested, with at least one notable peak. Samples collected during the second period had a lower overall water uptake as compared to those collected during period 1, even to the extent of having apparent negative values. The samples collected during the second period also showed greater variation between samples than those collected in the first period. As these samples were collected in the same location and rooms, it is likely that these differences arose due to different environmental conditions during the sampling periods. In a recent study, we have noted different water uptake by outdoor urban grime during different seasons^50^. Grime collected during the summer showed the most water uptake, followed by spring, and grime collected in winter showed the least uptake^50^. The results obtained during the present study are consistent with those findings. Samples collected during the summer period (July to September) showed greater water uptake than those collected during the fall/winter (October to December). This is likely due to air exchange between the indoors and outdoors, especially since it is more likely that windows would be open in the summer as compared to the winter, leading to increased ventilation. However, the difference in overall water uptake between the summer and fall/winter periods observed here is less significant than the summer-winter difference reported in ref.^50^. This observation could reflect the somewhat more controlled nature of the indoor environment. For example, surface and air temperature could impact both the chemical reactivity of surfaces and the partitioning behaviour of surface film constituents^6,19^. The indoor environment could be expected to vary less in temperature than outdoors, as heating and cooling are employed to offset significant fluctuations. Future studies could collect films concurrently indoors and outdoors over the course of one year in order to determine the dual impacts of ventilation and seasonality on water uptake by indoor films.

**Figure 4:**
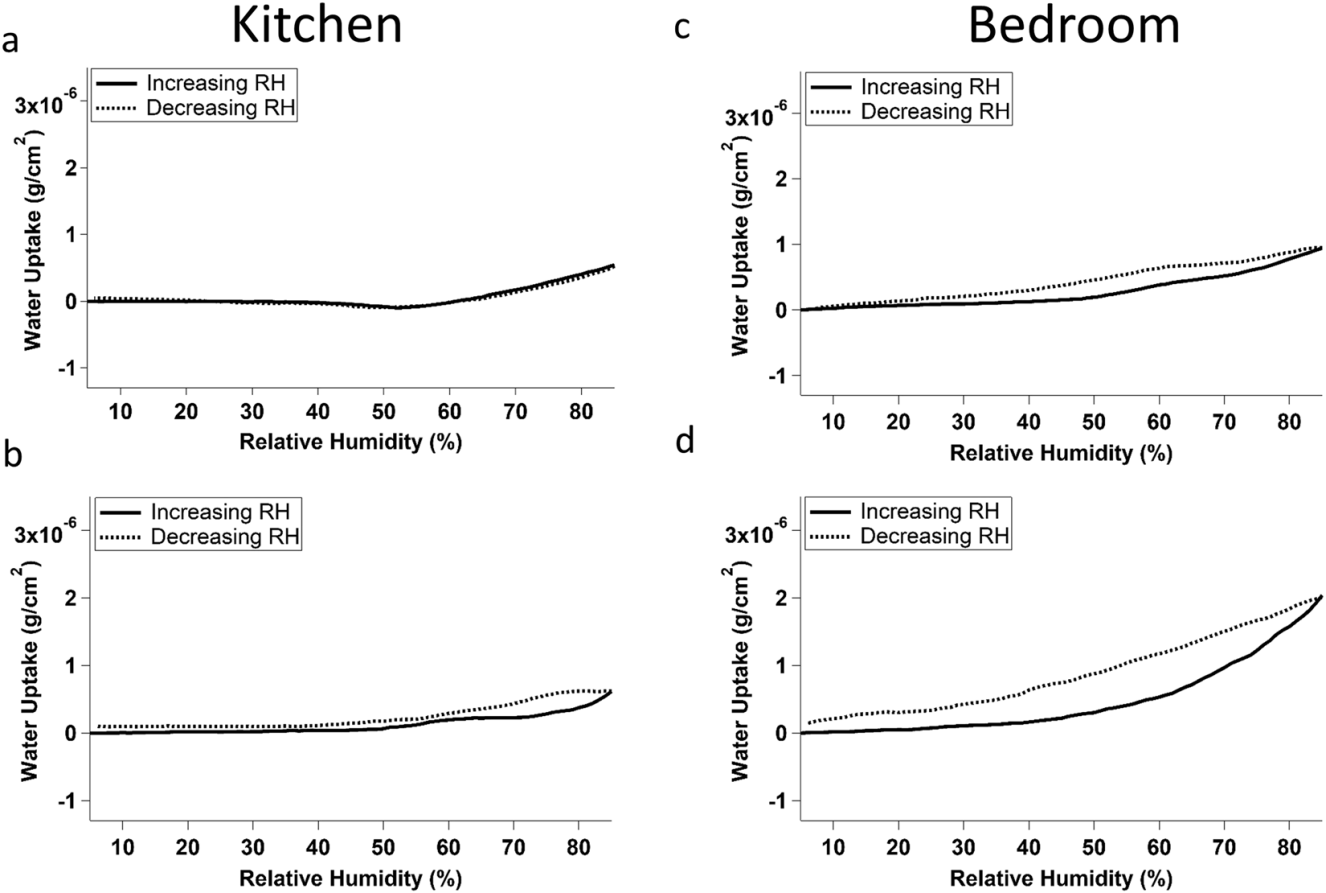
Averaged water uptake curves for 4 film samples collected in the kitchen (**a**,**b**) and bedroom (**c**,**d**) of Location 2 between December 21, 2018 and February 21, 2018. Solid lines indicate increasing relative humidity and dashed lines indicate decreasing relative humidity.

Figure 4 shows the water uptake curves of samples deployed between December 21^st^, 2018 – February 21^st^, 2019 in Location 2 in a kitchen and bedroom. In the kitchen, very little water uptake was observed below 50% RH and a small, smooth increase on mass was occurred between 50% and 85% RH. Almost no hysteresis was observed for either of the samples. The bedroom samples showed relatively smooth uptake across the entire range of RHs studied, and a small amount of hysteresis was observed between increasing and decreasing the RH in the cell.

When we compare sampling periods 1 and 2 (Figs 2 and 3) to sampling period 3 (Fig. 4), we can see the significance of room use and ventilation to water uptake by indoor films. Samples collected during periods 1 and 2 were collected in Location 1, whereas sampling period three was conducted in Location 2. Most of the samples collected in Location 1 showed the novel peak behaviour discussed above, suggestive of heterogeneity in the location of water uptake (leading to droplets), or phase transitions within the surface film. In contrast, those collected in location two had smooth uptake curves. This very different behaviour could be due to a number of room use factors, including the presence of pets in Location 1 and the cooking of meat in Location 2. The fats released by cooking meat in location two could have contributed to the lower water uptake observed in that location. Overall, water uptake of the samples collected in Location 2 (especially those collected in the bedroom) more closely resembles that of outdoor grime than the samples collected in Location 1 do.

The air exchange between sampling rooms was also very different in the two locations. In Location 1 the living room and kitchen were contiguous with only a hallway between them. In Location 2 the kitchen and bedroom are located on different floors. This difference in air exchange could explain why the samples collected in the kitchen and living room in Location 1 exhibit similar water uptake behaviour, while those collected in the kitchen and bedroom in Location 2 exhibit different water uptake behaviour. While the room use in both locations varies, the decreased air exchange between the two rooms in Location 2 means that the films which develop in each are more different from one another than are those in Location 1. In order to investigate these effects further, future studies should concurrently collect films in a variety of dwellings and in a various rooms within those dwellings.

### Ionic compositions of indoor films

**Figure 5:**
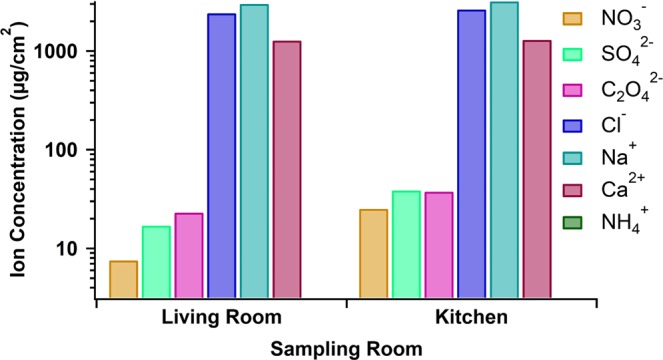
Blank-subtracted anion and cation concentrations in films collected on glass beads in Location 1 between November 19^th^, 2018 and February 6^th^, 2019.

In Fig. 5 we show the ionic compositions of indoor surface films collected in a living room and kitchen location in Location 1 between November 19^th^, 2018 – February 6^th^, 2019. All ions except ammonium were detected in both rooms sampled, and were present in higher surface concentrations in the kitchen. The values for the ionic surface concentrations are displayed in Table 1. It should be noted that due to limitations in sample size, each of the samples was only measured once for anions and once for cations. As such, we do not report a standard deviation in these measurements. These data are preliminary, as only one sample was able to be obtained from one location and one sampling period. However, much can be learned from these initial findings.

First, we note that nitrate, sulfate, oxalate, chlorine, sodium, and calcium were all present in films collected in both the living room and kitchen, while ammonium was not detected in either location. This is consistent with previous measurements of outdoor grime, where little if any ammonium was present in the grime despite its presence in particulate matter^50,51^. Previous studies have detected both gaseous NH_3_ and particulate NH_4_^+^ indoors, implying that ammonium could be expected to be found in indoor grime as well^36,61^. Several explanations have been proposed as to the depletion of ammonium from outdoor grime, including evaporation of ammonium nitrate, chemical exchange between sodium chloride and ammonium nitrate to form sodium nitrate, exchange between gas-phase hydrochloric acid and ammonia, or a biological conversion of ammonium to nitrate^51,56,62,63^. Any of these processes could be occurring indoors, and further investigation is required in order to determine which process is dominant in the indoor environment.

The overall concentrations of ions are in the films were between 2 and 4 orders of magnitude higher than those measured by outdoors in Toronto in December 2014^50^. This could be explained by a longer sampling period of 73 days as compared to the 31 days, and the different surface area shape of the beads as opposed to flat glass which could alter the physical accumulation of the film. However, they are also higher than the ion concentrations measured in outdoor grime in Leipzig, Germany by 1–4 orders of magnitude, despite the sampling time in that experiment being only 30 days shorter^51^. This could suggest greater accretion of ions on indoor surfaces as compared to outdoors. In addition, the ion ratios measured for the indoor site are lower in all cases than those reported by Baergen and Donaldson^50^. Those authors hypothesized that the 1:1 ratio of sodium to chloride they observed in urban grime during the winter period and in particles all year was due to the use of road salt during winter. However, the chloride to sodium ratio measured indoors during this study was roughly 0.5. This means that although a large portion of these ions could potentially be attributed to road salt, either excess sodium was deposited, or chloride was removed from the film.

Consistent with the facile air exchange between the sampling rooms in Location 1, discussed above in the context of water uptake, the surface concentrations of Na^+^, Cl^−^ and Ca^2+^ are very similar in those two rooms. This is consistent with all three ions being primarily of outdoor origin, being deposited indoors as a consequence of indoor-outdoor air exchange. Interestingly, higher surface concentrations of the remaining ions were measured in the kitchen compared to those collected in the living room.

Table 2 compares the mole ratios of ion surface concentrations within the films to the mole ratios measured in outdoor grime in Toronto by Baergen and Donaldson in December 2014^50^. The ratios of nitrate to sulfate and nitrate to calcium are both higher in the kitchen as compared to the living room, as is the overall oxalate mole fraction. The ratio of chloride to sodium is constant at roughly 0.5 in both the two rooms. In every case these ion ratios were lower than those observed in outdoor grime during winter^50^.While previous studies have shown more surface roughness and film coverage on kitchen samples as opposed to those collected in other rooms, this is the first example of higher ion concentrations on surfaces in the kitchen environment. Since facile air exchange between the rooms is demonstrated by the similar surface concentrations of Na^+^, Cl^−^ and Ca^2+^, the higher kitchen concentrations are probably indicative of the influence of highly local chemical processes. In particular, the significantly higher surface concentration of nitrate in the kitchen is consistent with cooking activities forming oxidized nitrogen oxides. The overall ionic ratio of oxalate in the film follows the same trend. Altogether, these observations imply that the accumulation and processing of ions within the indoor surface film differs according to location. This could be due to differences between the two locations with respect to light exposure, room use, and ventilation. We note that, despite these higher ionic concentrations measured for the kitchen samples, the water uptake curves in the two sample rooms of Location 1 were similar. This suggests that either the ions were not controlling water uptake by the films, or that this level of discrepancy in ion concentration was not enough to significantly alter the water uptake behaviour.

**Table 2:** Ratios of ions in indoor films collected in Location 1 between November 19th, 2018 and February 6th, 2019 compared to those reported by Baergen and Donaldson^50^.

Sample	NO_3_^−^: SO_4_^2−^	NO_3_^−^: Ca^2+^	Na^+^: Cl^−^	C_2_O_4_^−^: All Ions
Living Room	6.90 × 10^−1^	3.85 × 10^−3^	5.21 × 10^−1^	1.13 × 10^−3^
Kitchen	1.01	1.25 × 10^−2^	5.36 × 10^−1^	1.74 × 10^−3^
Baergen and Donaldson^50^	2.36	2.05	9.30 × 10^−1^	4.52 × 10^−3^

The results of this study indicate that indoor surface films have significantly different water uptake behaviour than outdoor grime, in spite of having a similar ionic composition. It thus is likely that the chemistry which occurs on the surface will differ from that occurring on outdoor urban grime. Indoor air can vary significantly in terms of relative humidity over short time scales, for example when a shower or a humidifier is turned on. It is possible that the unique water uptake indoor grime synergises with the physical and chemical properties of the substrate beneath to create novel chemical behaviours. As such, indoor deposits present a complex area of further study in the field of indoor heterogeneous chemistry.

## Methods

### Water uptake experiments

A quartz crystal microbalance (QCM) was used to measure water uptake by indoor surface films. The QCM consists of a piezoelectric oscillator, onto which can be placed crystals with specific fundamental frequencies. Previous studies have used QCM instruments to investigate the water-uptake behaviours of various chemical surfaces, including a variety of pure organics^46,47,50,64^. As mass is added onto the crystal, the frequency of vibration will vary linearly as a function of the Sauerbrey equation^47^:*Δf* = −*CΔm*, where m is the mass deposited, C is a proportionality constant of the crystal, and f is the frequency of vibration. This relationship holds for small changes in mass, and a maximum frequency change of 1% of the fundamental frequency^47,60^. The crystals used in this series of experiments were gold-plated quartz crystals 1.397 cm in diameter, with fundamental frequencies of 6 MHz and sensitivity parameters of 8.147 × 10^7^ Hz∙cm^2^∙g^−1^ ^47^.

The QCM apparatus was placed inside a Plexiglas cell which was connected to two mass flow controllers as shown in Fig. S4 in the Supplementary Information. Air Grade Zero 2 (Linde Canada) was either flowed directly through the tubing to reach the cell or was humidified through a bubbled filled with 18 MΩ∙cm deionized water. The relative flow through each mass flow controlled was adjusted throughout the experiment such that the relative humidity within the chamber varied from 5% to 85% at a rate of 1% per minute. During the experiment, the change in oscillator frequency for each percentage change in relative humidity was recorded, and the change in mass was calculated from those values.

Crystal blanks were obtained by cleaning the crystal with hexanes and methanol, followed by measurement over the 5% to 85% RH range with the QCM instrument. Blanks were subtracted from the collected film samples in order to account for water uptake onto the clean crystals themselves. In order to measure the water uptake of real indoor films, four crystal samples were deployed horizontally in occupied Toronto homes (two in the kitchen and two in a different room depending on location). Location 1 is located in the midtown of Toronto, on the third floor of a 4-storey apartment complex. There are two occupants and two cats within the dwelling on a regular basis. Location 2 is located in the East end of Toronto ~8 km away from Location 1. It is a 3-storey semi-detached house with 2 occupants and no pets. Table 3 outlines relevant parameters for each set of deployments.

**Table 3:** Relevant details for indoor grime collection on QCM crystals.

Assigned Name	Date	Location	Rooms Studied
Period 1	July 25, 2018-September 21, 2018	Location 1	Kitchen and Living Room
Period 2	October 11, 2018-December 11, 2018	Location 1	Kitchen and Living Room
Period 3	December 21, 2018-February 21, 2018	Location 2	Kitchen and Bedroom

### Film composition experiments

The collection and extraction procedure for ions in indoor films was adapted from the method reported by Baergen *et al*.^51^. In order to collect indoor films, two samples of ~30 g of 3 mm diameter soda-lime glass beads were placed in a single layer in petri dishes and were deployed in the kitchen and the living room of an occupied dwelling (Location 1) between November 19th, 2018 and February 6th, 2019. Prior to deployment, the beads were rinsed 10x with tap water and 5x with 18 MΩ∙cm DI water, and left to soak overnight in DI water. They were then rinsed 5x with dicholoromethane and baked at 200 °C for 24 hours.

In order to extract ions from the films, the beads were transferred into a Nalgene bottle containing 30 mL DI water and the bottle was shaken for 5 min. The aqueous extract was decanted and filtered through a 0.45 μm syringe filter to remove insoluble materials. The resulting solutions were analyzed using ion chromatography to measure NO_3_^−^, SO_4_^2−^, C_2_O_4_^2−^, Cl^−^, Na^+^, Ca^2+^, and NH_4_^+^. The instrument used was a Metrohm 930 Compact IC Flex fitted with a conductivity detector. For cation analysis a Metrosep C6 150 × 4 mm column was employed and 0.9 mL·min^−1^ of 3.4 mM HNO_3_ was used as eluent. For anion analysis a Metrosep A Supp 5 150 × 4 mm column was used as well as a Metrohm Suppressor Module with 100 mM H_2_SO_4_ IC Suppression Regeneration Solution and 0.7 mL·min^−1^ of 1 mM NaHCO_3_ and 3.2 mM Na_2_CO_3_ were used as eluent.

Blanks were obtained by performing an extraction and analysis on beads which had been cleaned in the manner described previously. The blank values were then averaged and subtracted from all samples. In order to determine the extraction efficiency, a second extraction and analysis was carried out on the sample collected in the kitchen. NO_3_^−^, SO_4_^2−^, Cl^−^, Na^+^, and NH_4_^+^ were all below blank levels in the second extract, and C_2_O_4_^2−^ and Ca^2+^ showed 9% loss and 13% loss respectively. Representative calculations for the ion concentrations can be found in the Supplementary Information.

The datasets generated during and/or analysed during the current study are available from the corresponding author on reasonable request.

## Supplementary information

Supplementary information for Schwartz-Narbonne & Donaldson
