# Supplementary material for: Water uptake by indoor surface films
Source: Sci Rep. 2019 Jul 31;9:11089. doi: 10.1038/s41598-019-47590-x (PMC6668427; doi:10.1038/s41598-019-47590-x)
Supplement: Supplementary file 1 — Supplementary information for Schwartz-Narbonne & Donaldson [file 41598_2019_47590_MOESM1_ESM.docx]

**Supplementary Information for Water uptake by indoor surface films**

Heather Schwartz-Narbonne and D. James Donaldson


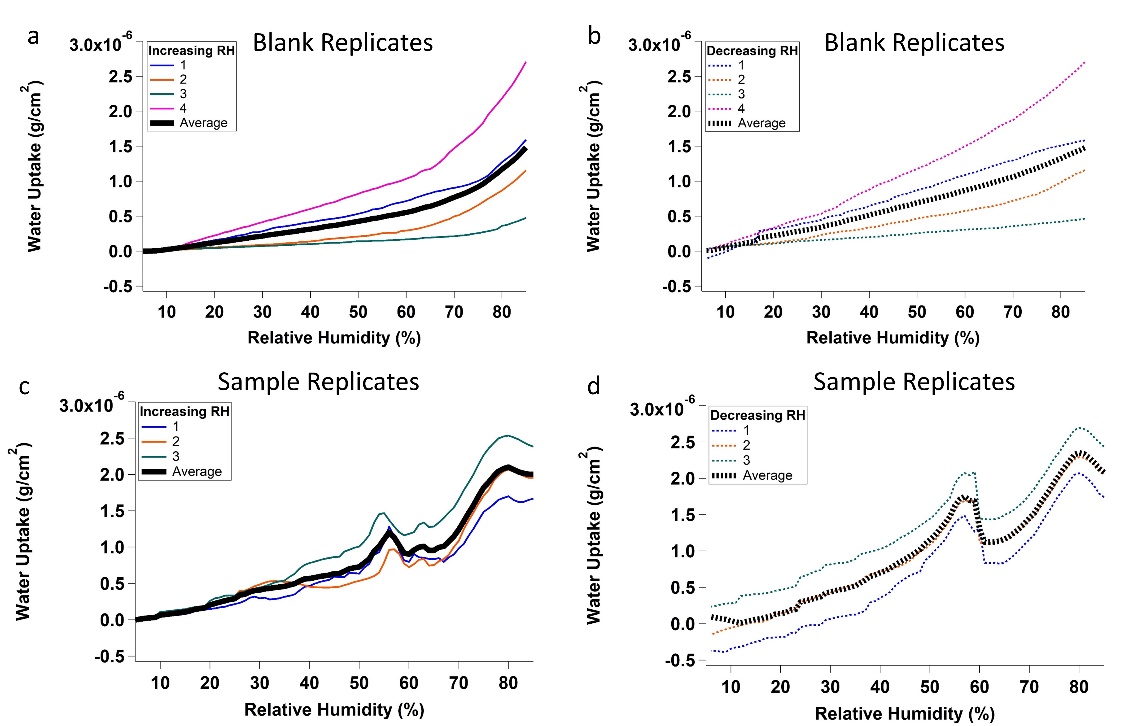


Figure S1: Representative replicates of blank crystal (a and b) and sample crystal (c and d) experiments. Solid lines indicate increasing relative humidity and dashed lines indicate decreasing relative humidity. The averaged curves are shown as a thick black line. Although the overall water uptake of both the blanks and samples vary in terms of absolute values, the overall shape of the curves is maintained between experimental repetitions.


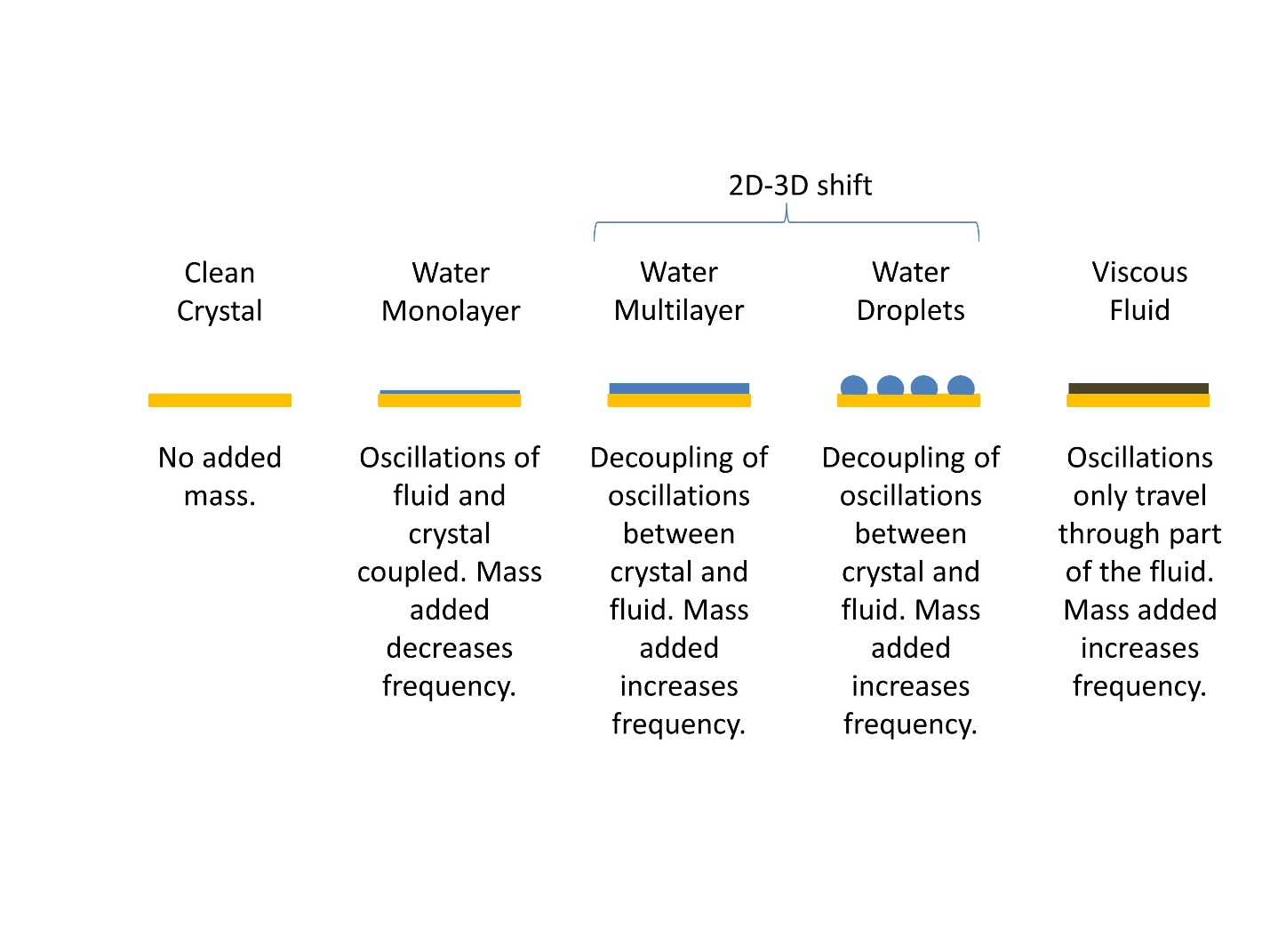


Figure S2: A graphic illustrating the different possible physical phenomena by which an increase in mass on a QCM crystal could result in an increase in oscillation frequency.


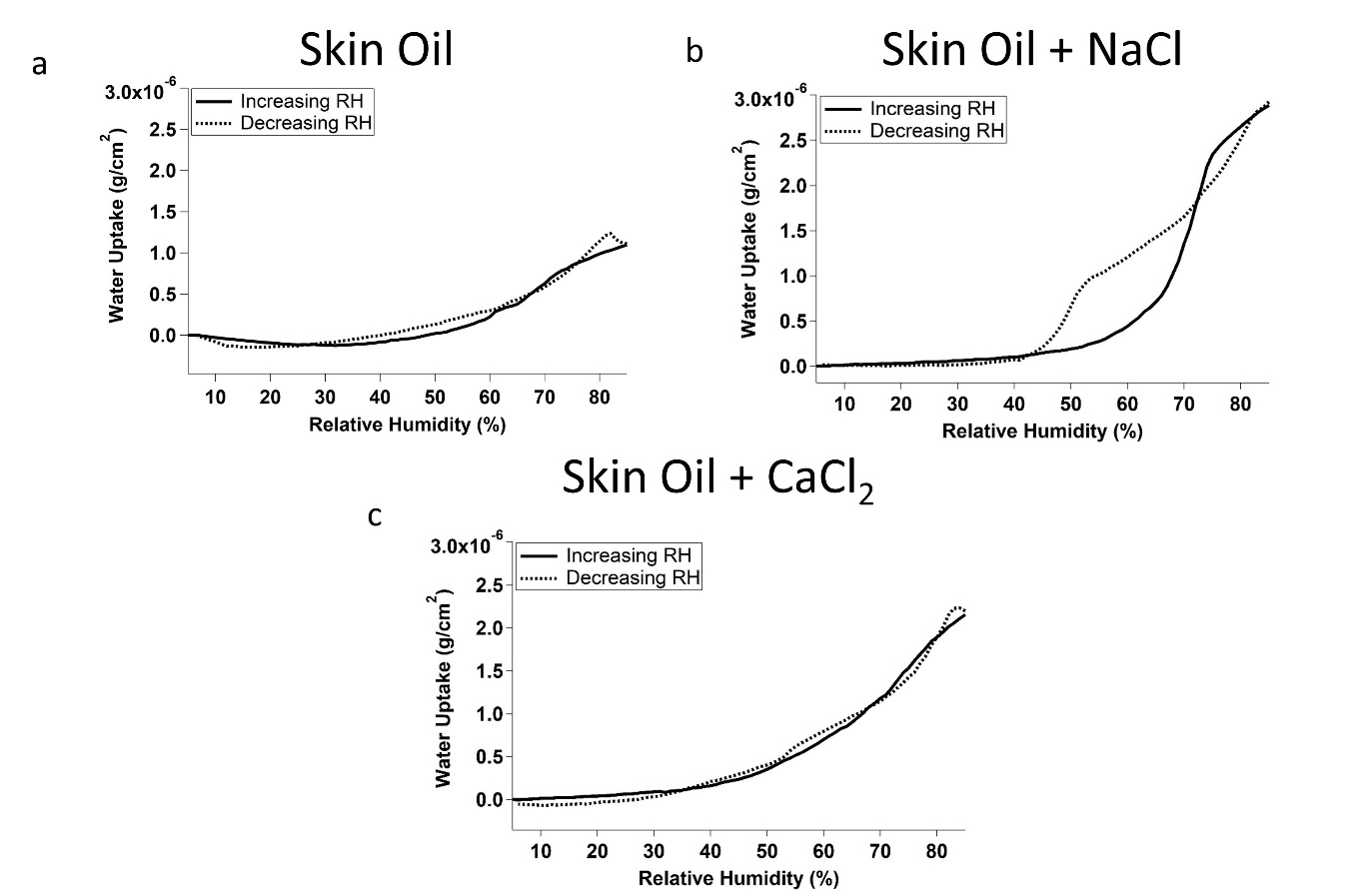


Figure S3: Averaged water uptake curves for laboratory film tests consisting of skin oil (a), skin oil combined with sodium chloride (b) and skin oil combined with calcium chloride (c). The solid lines indicate increasing relative humidity and dashed lines indicate decreasing relative humidity.

A reproducible film of skin oil was produced by wiping of the QCM crystal surface twice with the thumb of a freshly washed hand. In the case of films containing salts, sodium chloride or calcium chloride were crushed and placed on the crystal after the skin oil was applied.

Skin oil films displayed moderate water uptake, which likely reflects the presence of saturated and oxidised organic molecules. When sodium chloride was applied to this film, the uptake increased significantly, and efflorescence and deliquescence points were observed. When calcium chloride was applied to the film the overall uptake was increased but there was no evidence of efflorescence or deliquescence.


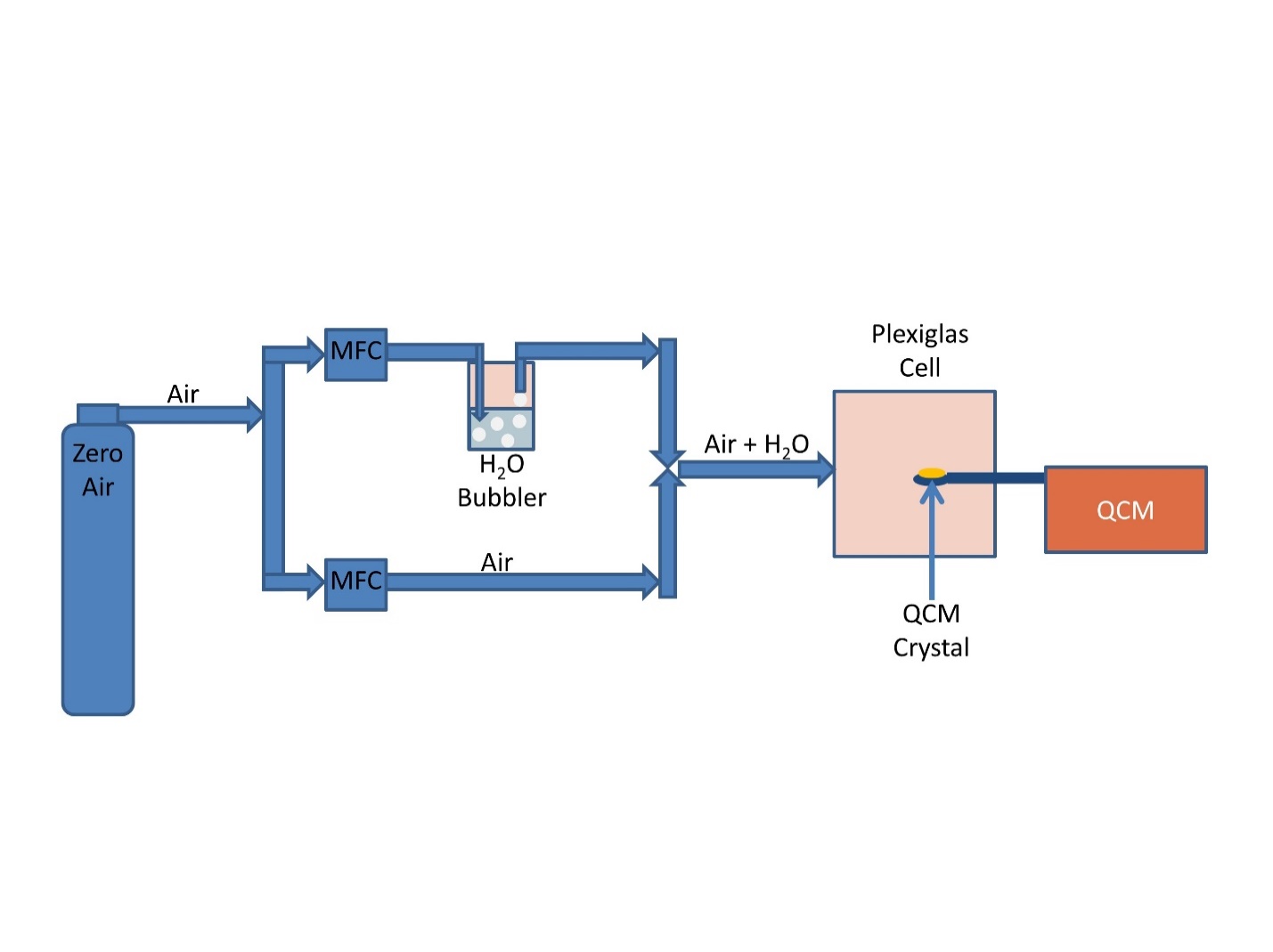


Figure S4: Experimental set-up for the water uptake experiments, using the quartz crystal microbalance.

*Ion Analysis*

$$y=mx+b (obtained from calibration curve for ion)$$

$$\frac{y_{sample}-b}{m}=x_{sample}(\frac{\mu g}{g})$$

$$Total Mass in Extract (\mu g)= x_{sample}\left( \frac{\mu g}{g} \right)\cdot30 g H_{2}O$$

$$Number of Beads= \frac{mass of beads (g)}{density of beads \left( \frac{g}{{cm}^{3}} \right)\cdot Volume ({cm}^{3})}$$

$$Total Surface Area ({cm}^{2})=Number of Beads\cdot Surface Area of One Bead ({cm}^{2})$$

$$Final Ion Concentration\left( \frac{\mu g}{{cm}^{2}} \right)= \frac{Total Mass in Extract (\mu g)}{Total Surface Area ({cm}^{2})}$$

Table S1: Measured concentrations of ions in indoor films collected in Location 1 between November 19th, 2018 and February 6th, 2019.

| Concentration (ug/cm^2) | | | | | | | |
| --- | --- | --- | --- | --- | --- | --- | --- |
| Sample | NO_3_^-^ | SO_4_^2-^ | C_2_O_4_^2-^ | Cl^-^ | Na^+^ | Ca^2+^ | NH_4_^+^ |
| Living Room | 7.57 | 17.00 | 23.05 | 2412.96 | 3003.72 | 1272.08 | 0 |
| Kitchen | 25.17 | 38.72 | 37.64 | 2633.24 | 3184.44 | 1298.52 | 0 |
| Kitchen (Extraction 2) | 0 | 0 | 3.17 | 0 | 0 | 207.90 | 0 |
